# Nitrogen Use Efficiency, Allocation, and Remobilization in Apple Trees: Uptake Is Optimized With Pre-harvest N Supply

**DOI:** 10.3389/fpls.2021.657070

**Published:** 2021-05-31

**Authors:** Bi Zheng Tan, Dugald C. Close, Peter R. Quin, Nigel D. Swarts

**Affiliations:** Tasmanian Institute of Agriculture, College of Sciences and Engineering, University of Tasmania, Hobart, TAS, Australia

**Keywords:** nitrogen use efficiency, 15N, remobilization (nitrogen), partitioning (nitrogen), application timing, storage (nitrogen), nitrogen uptake

## Abstract

Optimizing the utilization of applied nitrogen (N) in fruit trees requires N supply that is temporally matched to tree demand. We investigated how the timing of N application affected uptake, allocation, and remobilization within 14-year-old “Gala”/M26 apple trees (*Malus domestica* Borkh) over two seasons. In the 2017–2018 season, 30 g N tree^−1^ of 5.5 atom% ^15^N–calcium nitrate was applied by weekly fertigation in four equal doses, commencing either 4 weeks after full bloom (WAFB) (pre-harvest) or 1-week post-harvest, or fortnightly, divided between pre- and post-harvest (50:50 split). Nitrogen uptake derived from fertilizer (NDF) was monitored by leaf sampling before whole trees were destructively harvested at dormancy of the first season to quantify N uptake and allocation and at fruit harvest of the second season to quantify the remobilization of NDF. The uptake efficiency of applied N fertilizer (NUpE) was significantly higher from pre-harvest (32.0%) than from the other treatments (~17%). The leaf NDF concentration, an indicator of N uptake, increased concomitantly only when pre-harvest N was applied. Pre-harvest treated trees allocated more than half of the NDF into fruit and leaves and stored the same amount of NDF into perennial organs as the post-harvest treatment. Subsequent spring remobilization of NDF was not affected by the timing of N fertigation from the previous season. A seasonal effect of remobilization was observed with a decrease in root N status and a reciprocal increase in branch N status at fruit harvest of season two. These findings represent a shift in the understanding of dynamics of N use in mature deciduous trees and indicate that current fertilizer strategies need to be adjusted from post-harvest to primarily pre-harvest N application to optimize N use efficiency. This approach can provide adequate storage N to support early spring growth the following season with no detriment to fruit quality.

## Introduction

Deciduous fruit trees internally cycle and reuse stored nitrogen (N) from one growing season to the next. Nitrogen is withdrawn from leaves prior to leaf abscission and stored in roots, branches, and the trunk for over-wintering (Millard and Thomson, 1989). Stored N is remobilized for early spring growth and makes an important contribution to the seasonal N budget for the fruit tree crops (Masclaux-Daubresse et al., 2010). Seasonal N requirements are thus comprised of internal stored N (Chapin III et al., 1990) and N taken up by roots during the growing season (Millard and Grelet, 2010). In a commercial orchard, root N uptake is predominantly from the application of N fertilizer, which has been demonstrated to be vital to supply the demands of vegetative and reproductive growth (Klein et al., 1989; Maathuis, 2009). However, N fertilizer can be oversupplied in production systems due to the perceived cost-effectiveness of achieving increased yield per unit area (Drake et al., 2002; Neilsen et al., 2009). In apple orcharding, this can cause reductions in fruit quality (Carew, 2000) and low use efficiency of N resources (Neilsen et al., 2001a), contributing to the pollution of underground water supplies (Neilsen and Neilsen, 2002), and emissions of the potent greenhouse gas nitrous oxide (Freney, 1997; Swarts et al., 2016). Nitrogen is also commonly applied post-harvest either *via* fertigation or the application of foliar-applied urea, with the aim of increasing N reserves in storage organs (i.e., buds, spurs, and roots) and for faster decomposition of the leaf litter material (Han et al., 2011). However, there is currently limited knowledge of how post-harvest N application contributes to the current season N storage and N sink for subsequent spring remobilization in mature apple trees (Millard and Grelet, 2010) and its effectiveness in improving fruit productivity and quality. In contrast, matching N supply with tree uptake requirements optimizes marketable yield and minimizes environmental impact (Gebbers and Adamchuk, 2010), a primary objective of precision agriculture.

Nitrogen uptake efficiency (NUpE), defined here as “the proportion of fertilizer N recovered in tree organs when applied in a growing season” (Neilsen et al., 2001b), has also been referred to as apparent fertilizer N recovery (Benincasa et al., 2011). Cassman et al. (2002) defined a similar term, fertilizer N recovery efficiency (NRec) for annual crops, which is modified for this study as the proportion of fertilizer N recovered in tree organs either in the year of application or any single subsequent year. Hill-Cottingham and Lloyd-Jones (1975) showed that NUpE for young apple trees was either about 40 or 16% when N fertilizer was applied in the early spring or soon after the fruit harvest, respectively. Low NUpE in perennial tree crops has been attributed to the sparse distribution of root systems (Neilsen and Neilsen, 2002) and/or mismatched rate and timing of applied N to tree demand (Hill-Cottingham and Lloyd-Jones, 1975; Aguirre et al., 2001; Neilsen et al., 2001a; Drake et al., 2002).

The allocation of fertilizer N throughout a fruit tree is strongly influenced by the timing of its application. Toselli et al. (2000) showed that the majority of spring applied N in apple trees was allocated into fruit and leaves (10.2 and 12.3% of total N, respectively), and a relatively small amount was stored in roots (1.6% of total N), whereas most of the summer applied N was allocated into the perennial structures such as roots and 2- to 4-year-old wood (18.0 and 12.9% of total N, respectively). Furthermore, an increase in stored N in the roots and wood of newly planted apple trees can influence the manner of N remobilization in the subsequent season. Neilsen et al. (2001a) showed that trees supplied with N in spring, 2–8 weeks after planting in the following season remobilized from storage 7% more N into fruitlets (18% of total remobilized N) and 6% less N into leaves (77% of total remobilized N) than those with N applied in summer, 8–14 weeks after planting. Although N storage and uptake in young trees have been investigated (Dong et al., 2001; Neilsen et al., 2001a), a key knowledge gap exists in the uptake, allocation, and internal cycling of N in commercially managed mature apple trees. The fruit yield and quality associated with different N uptake and allocation from different N application timings are especially important to the sustainability and profitability of fruit production. This knowledge is key to establishing an accurate N budget and recommendations for precise N management in orchards.

In earlier research, Scandellari et al. (2008) conducted a comprehensive 6-year study of the macronutrient (N, P, K, Ca, Mg, and S) and micronutrient (B, Fe, Mn, Zn, and Cu) budget in apple production through the destructive harvest of whole trees, followed by a mass balance of macronutrient content, to determine the nutrient supply, storage, and allocation, with the use of conventional mineral (N, P, K, and Mg) fertilizer. However, the study neither differentiated between nitrogen derived from fertilizer (NDF) and preexisting N in the soil nor investigated internal tree N remobilization. Applied N can be traced throughout the tree through the use of an N source enriched with the stable isotope ^15^N (Hauck and Bremner, 1976; Dong et al., 2002). This approach has been used to quantify fertilizer N uptake in deciduous fruit tree crops, including apples (Neilsen et al., 1997; Guak et al., 2003; Zhang et al., 2012), pears (Quartieri et al., 2002), cherries (San-Martino et al., 2010; Rivera et al., 2016), and evergreen fruit tree crops, such as citrus (Martínez-Alcántara et al., 2011). For example, when 10-year-old field-grown apple trees were supplied with ^15^N-labeled fertilizer at bud burst and destructively sampled periodically, the NUpE of 9.9 to 12.2% was quantified over two seasons and the majority of NDF was allocated to perennial organs (Zhang et al., 2012). A similar approach, with the inclusion of successive xylem sap sampling, was deployed on 2-year-old apple trees (Guak et al., 2003) demonstrating that leaf growth was mostly supported by remobilized N, and root uptake did not commence until 14 days after remobilization had begun.

The majority of horticultural studies using the ^15^N isotope tracing method have been conducted in pot trials using young or newly planted trees, whereas the requirement for N varies with tree age and phenological stages (Schenk, 1996) and more significantly, in commercial settings with higher crop loads. A challenge to the success of this technique *in situ* has been the recovery of ^15^N in plant material from a large tree structure for complete mass balancing. However, the utilization of dwarfing rootstocks in modern apple orchards and the use of netting to capture all shed materials has made recovery of the whole tree structure a more practical procedure.

This study aimed to address the knowledge gaps identified above in a high-density commercial orchard through the destructive sampling of whole trees at two different timings. Specifically, we aimed to (1) quantify apple tree N uptake and NUpE under early (pre-harvest), late (post-harvest), and split (50:50 split) N application, (2) determine the impact of N application timing on N allocation and storage, (3) quantify the remobilization of tree-stored N in the season following that of the applied-N treatments, and (4) assess the impact of N application timing on attributes of fruit quality.

## Materials and Methods

### Experiment Site and Trees

The experiment was conducted from August 2017 to June 2019 over two growing seasons in a commercial orchard located at Plenty in the Derwent Valley, southern Tasmania (42°44′31″S, 146°58′22″E), Australia. Mean maximum and minimum temperatures are 11.5 and 1.5°C in the winter months and 24 and 10°C in the summer months, respectively. The mean annual rainfall is 572.2 mm, with summer being the driest of a fairly even seasonal distribution. The orchard block was planted on a Dermosol with a sandy loam A horizon ~40 cm deep. The apple trees were 14-year-old “Gala” cultivar grown on M26 rootstock planted in 2005 at a density of 1,667 tree ha^−1^, trained to a central leader system supported by a trellis. A 1.0-m wide weed-free strip along the tree line was maintained with the applications of glufosinate-ammonium herbicide. The trees were subjected to commercial management practices prior to the experiment. Trees were irrigated throughout the growing season using microjet sprinklers (50 L h^−1^) for 2–3 h when low soil moisture levels were detected by soil moisture probes.

### Experimental Treatments and Design

The experiment was established over three sampling rows, each containing a total of 33, 36, and 15 trees, respectively, with two buffering rows of trees between each. The first, second, and third sampling rows contained 11, 12, and 5 sampling trees, respectively. Each sampling tree was buffered by one non-trial tree on each side. A set of 16 sampling trees from the first and third sampling rows, deployed in a complete randomized design, received all of four fertigation treatments replicated four times. The 12 trees from the second sampling row deployed a randomized complete block design with the 50:50 split treatment excluded, and the remaining treatments replicated four times. Different experimental designs were used for each set of tree plots as there were a limited number of trees per row that had soils of a similar type and profile.

The experimental N treatments consisted of the application of 5.5 atom% ^15^N-labeled calcium nitrate (N_f_) by fertigation at the rate of 30 g N tree^−1^ at different timings. The timings were pre-harvest (spring—commencing on November 7, 2017, 4 WAFB), post-harvest (autumn—commencing on March 21, 2018, 1-week post-harvest), 50:50 split (fertigation was equally divided to pre- and post-harvest), or control where N fertigation was excluded (Table 1). The pre- and post-harvest treatments were divided equally over four successive weekly applications. The 50:50 split treatment was divided equally over two pairs of fortnightly applications, each commencing at the same time as the pre- and post-harvest treatments. All treatments were also applied to one buffer tree on either side of a sampling tree. Treatments were delivered *via* a fertigation system with electric pumps (Shurflo 4009-101-A87, Pentair, Costa Mesa, CA, United States) at a flow rate of 11.3 L min^−1^, and four pressure compensated drippers (2 L h^−1^) placed 20 cm away from the tree trunk at the corners of a square grid formation. For each fertigation event, water was pumped through the fertigation line for 10 min, followed by 30 min of N_f_ solution at 1.875 g N L^−1^, and finished by 10 min of flushing with water. No further N was applied for the duration of the trial, to allow an examination of the fate, in the 2018–2019 season, of N_f_ applied in the 2017–2018 season. All non-N nutrient application, pest, and weed control were carried out in line with a standard orchard practice. A timeline of key experimental events represented in weeks after full bloom relative to the tree phenological growth stage is presented in Table 1.

**Table 1:** Key management events and the respective growth stages over the 2017–2018 and 2018–2019 growing seasons, related to weeks after full bloom (WAFB).

**Experiment timeline and management schedule**		
**Growth stage**	**Time (WAFB)**	**Key management event**
**Season 1 (2017−2018)**		
Full bloom (October 10, 2017)	0	
Petal fall	4	Pre-harvest N application start
Early fruit set	7	Pre-harvest N application end
Fruit ripening	22	Fruit harvest and quality assessment
Leaf yellowing	23	Post-harvest N application start
Leaf fall	26	Post-harvest N application end
	30	Post-storage fruit quality assessment
Winter dormancy	36–37	Whole tree excavation
Winter dormancy	41	Soil sampling
**Season 2 (2018**−**2019)**		
Full bloom (October 8, 2018)	0	
Fruit ripening	20	Fruit harvest and quality assessment
Post-harvest	21	Whole tree excavation
	28	Post-storage fruit quality assessment

### Leaf, Fruit, Soil Sampling, and Whole Tree Excavation

Ten randomly selected fully grown bourse leaves were sampled weekly from the middle section of each tree, from 3 WAFB until leaf fall throughout the 2017–2018 growing season. Leaf samplings at 4 and 23 WAFB were completed 1 day after the application of treatments. Orchard netting was installed below each tree early in the season and lifted after fruit harvest (22 WAFB) to cover the whole tree canopy to ensure the capture of all leaves during autumn. All fruits were harvested at commercial maturity in both growing seasons on March 13, 2018 and February 28, 2019, respectively, to calculate total biomass and recovery of applied N_f_. A subsample of 50 fruits was used for fruit quality assessment, 25 at harvest, and the remainder at 8 weeks post-harvest, having been stored at ~2°C at normal atmosphere throughout that period.

Soil samples of 0–10 cm depth were taken at about the middle of dormancy on July 11, 2018, 14 weeks before bud break of the 2018–2019 season, using a step probe (2.2 × 10.0 cm cores). For each tree, two samples were taken from the soil within a 10 cm radius of two of the four fertigation drippers, another from midway between one of those drippers, and the remaining two from midway between those remaining two drippers. All four samples were homogenously mixed into one, air-dried until a constant weight, and then passed through a 2 mm sieve.

The set of 16 trees were destructively harvested at dormancy of the 2017–2018 season (June 2018) to examine the uptake, allocation, and storage of NDF in the 2017–2018 season. The set of 12 trees were destructively harvested 1 week after commercial fruit harvest of the 2018–2019 season (March 2019) to measure the contribution of stored and remobilized NDF to tree growth the subsequent season. First, the above-ground portions of the trees were removed to just above the graft union. The remaining tree structure including roots was then harvested by carefully digging the soil to 0.6 m depth and 1 m radius around the tree trunk. The remaining soil was thoroughly examined for any broken root material by two independent groups of people to ensure recovery of as much of the root systems as possible. Trees were separated into different organs of leaf, bud, spur, 1st-year wood, branch (≥2 years), trunk, coarse root (>1 cm), medium root (<1 cm and >2 mm), and fine root (<2 mm), before fresh weight and a representative subsample were taken for each organ. Subsampled organs and collected leaf samples were dried at 60°C until a constant weight was obtained, the ratio of dried-to-fresh weights for each subsample being applied to calculate the total dried weight of each harvested organ. Prior to the analysis, homogenized subsamples of plant material and sieved-soil samples were ground into a fine powder using a MM400 Ball Mill (Retsch, Haan, Germany).

### Total N and ^15^N Analysis

Samples were analyzed for N percentage and ^15^N atom percentage (^15^Napc) at the Central Science Laboratory, University of Tasmania. Stable N isotopes were analyzed using the flash combustion isotope ratio mass spectrometry (varioPyro cube was manufactured by Elementar, in Sydney, Australia. IsoPrime100 was manufactured by IsoPrime, in Cheadle, United Kingdom). Stable isotope abundances were reported in delta (∂) values as the deviations from conventional standards in parts per mil (‰) from the following equation:

$$\begin{array}{l} \partial^{15}\text{N}(‰)=[{(}^{15}\text{N}{/}^{14}\text{Nsample})/{(}^{15}\text{N}{/}^{14}\text{Nstandard})-1]\times \;1,000 \end{array}$$

δ^15^N values were reported relative to atmospheric air. Certified Reference Materials (USGS40, USGS41, IAEA-N1, and IAEA N2) were used to correct for instrumental drift and quality assurance purposes. As recommended by IUPAC (Coplen et al., 1992), the value of 272 was employed for ^14^N/^15^N of N_2_ in the air for the calculation of atom fraction ^15^N from measured δ^15^N values; the applied formula (Hauck, 1982) is valid for low enrichments (<5 atom %). Enriched laboratory standards were prepared from mixtures of enriched and natural abundance fertilizer and calibrated against international reference standard IAEA311. The analytical performance of the instrumentation, drift correction, and linearity performance was calculated from the repetitive analysis of these standards. The precision of the elemental data was 0.2%. For isotopic measurements, the precision was <0.06‰ up to the highest enrichment level. The ^15^N atom percentage was calculated from the measured δ^15^N values and the calibration curve produced from the enriched laboratory standards. Natural abundance (NA) of ^15^N used in the calculation was the ^15^Napc measured from the leaf sample prior to the application of enriched calcium nitrate, which was 0.3689 ± 6.67 × 10^−5^ at 3 WAFB (see Supplementary Table 1). The proportion of N within a plant organ that was derived from N_f_ is represented by NDF_organ_ and is given as:

$$\begin{array}{l} NDF_{organ}={(}^{15}Napc_{organ}-NA)/(N_{f}-\;NA); \end{array}$$

NDF_tree_ represents the sum of NDF of all organs, thus:

$$\begin{array}{l} {NDF}_{tree}=\sum \left({NDF}_{organ}\;\times \;dry\;weight\;of\;organ\right); \end{array}$$

NUpE represents NDF_tree_ at 2018 dormancy as a percentage of N_f_ applied, and NRec represents NDF_tree_ in the second season as a percentage of N_f_ applied (in the previous season). These were calculated with the formulae:

$$\begin{array}{l} NUpE\;=\;\frac{{NDF}_{tree}\;at\;2018\;dormancy}{N_{f}\;applied}\times \;100\\ NRec\;=\;\frac{{NDF}_{tree}\;at\;2019\;harvest}{N_{f}\;applied}\;\times \;100 \end{array}$$

The N status of the tree represents the total N component of the whole tree structure at the time of measurement, comprised of native N (non-fertilizer-derived N) and NDF. Native N and NDF, of both leaf foliage and fruit organs for the first season, and of fruit at harvest for the second season, were excluded from N status calculation—so that the overall N remaining within the tree system at the time of storage and after remobilization could be compared on a standardized basis.

### Fruit Quality Analysis

At fruit harvest, a subsample of 25 fruits from each treatment was taken for the assessment of fruit quality. The fruit was weighed on a GX-4000 laboratory balance (A&D, Tokyo, Japan) and its size was measured using Vernier calipers. Peel red color coverage was visually rated using a color chart ranging from 1 to 5, where 1 represented 0–20% red coverage over the fruit and successive ranks increased in 20% steps. Red color intensity was measured visually with a “Royal Gala” color chart (Enza, New Zealand) ranging from 1 to 11, where 1 represents light red and 11 represents a dark red color. Background color of peel where red pigmentation was not developed was visually assessed with a color chart ranging from 1 to 10, with the color graded from green (1), to light green (5), to yellow (10). A delta absorbance meter was used to assess the maturity of the fruit by measuring the chlorophyll-a in the fruit mesocarp (Costa et al., 2009). Peel blush color was assessed using a CR-400 Chroma Meter (Konica Minolta, Ramsey, NJ, United States) and reported in the CIE L^*^a^*^b^*^ color space (CIE, 2019), with an average of three random measurements from that area of each fruit. A thin slice of peel was removed on the blush side of each apple to measure flesh firmness using a GUSS Fruit Texture Analyzer (GUSS, Cape Town, South Africa). Juice was collected to measure total soluble solids (TSS) by using a Digital Refractometer (Atago Co., Tokyo, Japan). The fruit was then cut horizontally in half, and the cut surface of the bottom half was sprayed with iodine solution and left to dry for 5 min before the starch index was visually assessed against the Cornell starch–iodine index (1, full starch; 8, no starch) (Blanpied and Silsby, 1992).

### Statistical Analysis

Statistical analysis was completed using the R statistic package (Team, 2019). Data were subjected to either one-way or two-way ANOVA test or *t*-test assumption tests prior to the analysis. The one-way or two-way ANOVA and Tukey's HSD tests were used to compare the means of N application treatments at a 95% confidence level. An unpaired two-sample *t*-test was used to compare means of NDF allocation and N status of organs after remobilization between pre- and post-harvest N application treatments, and means of organ N status were pooled from pre- and post-harvest N treatment between season 1 and season 2.

## Results

### Nitrogen Uptake Efficiency and Tree N Status

**Figure 1:**
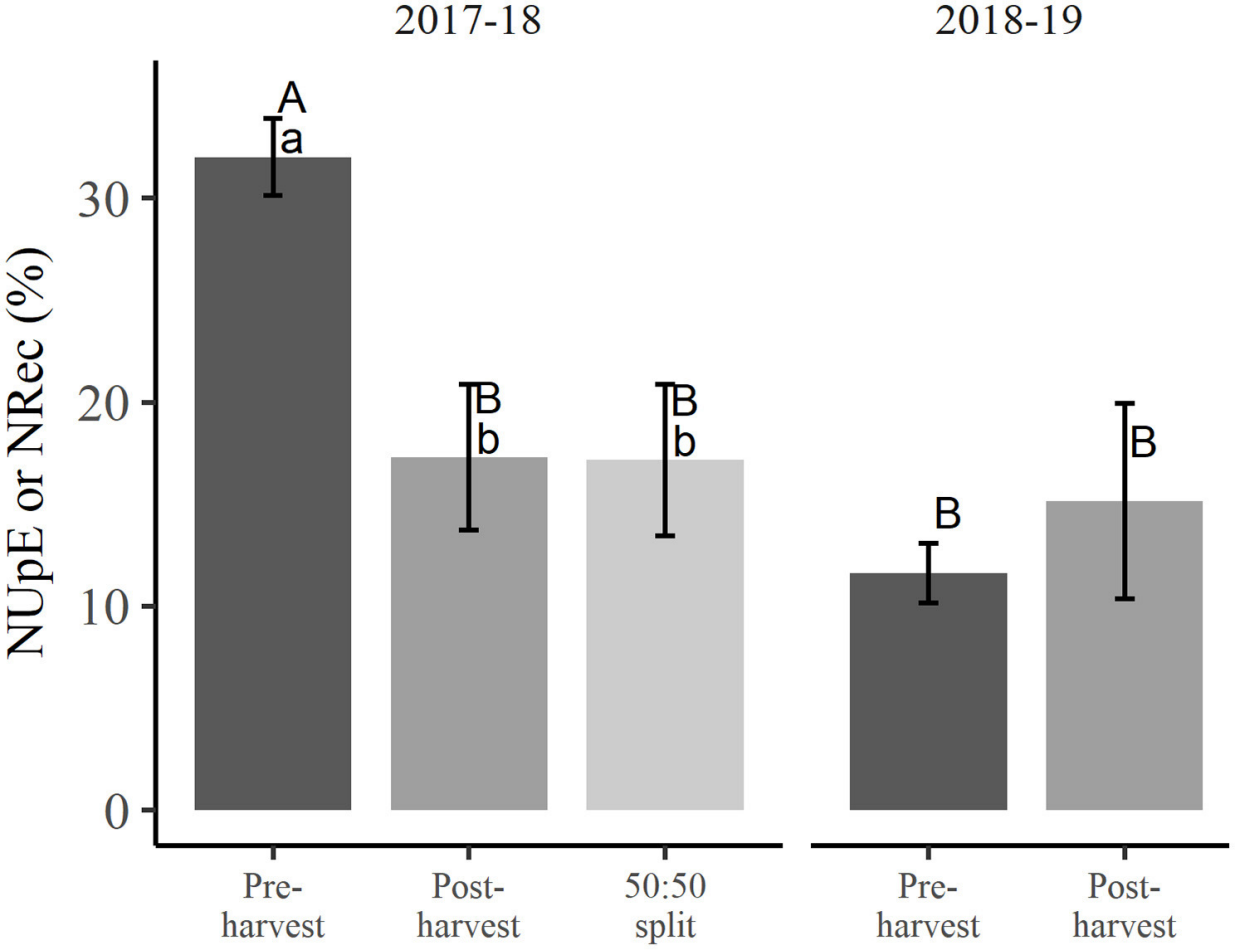
Nitrogen uptake efficiency (NUpE) in the 2017–2018 season and nitrogen recovery efficiency (NRec) in the 2018–2019 season following different N application treatments. Different uppercase letters denote significant interaction (*p* < 0.05) between treatments and seasons and different lowercase letters indicate significant differences (*p* < 0.05) between treatments within a season (error bars represent ±SEM, *n* = 4).

At winter dormancy following treatment applications in the first season, NUpE of 32.0% in the pre-harvest treated trees was significantly higher (*p* < 0.05) than for the post-harvest and 50:50 split treated trees, which had very similar NUpE of close to 17.2% (Figure 1). For the pre-harvest treated trees, the NDF taken up in the first season was significantly greater (*p* < 0.05) than NDF recovered (11.6% NRec) in the second season (Figure 1). The NRec of trees receiving post-harvest N treatment (15.1%) was not significantly different from either the NDF taken up in the previous season (NUpE 17.2%) or the NRec of pre-harvest treated trees (Figure 1).

**Figure 2:**
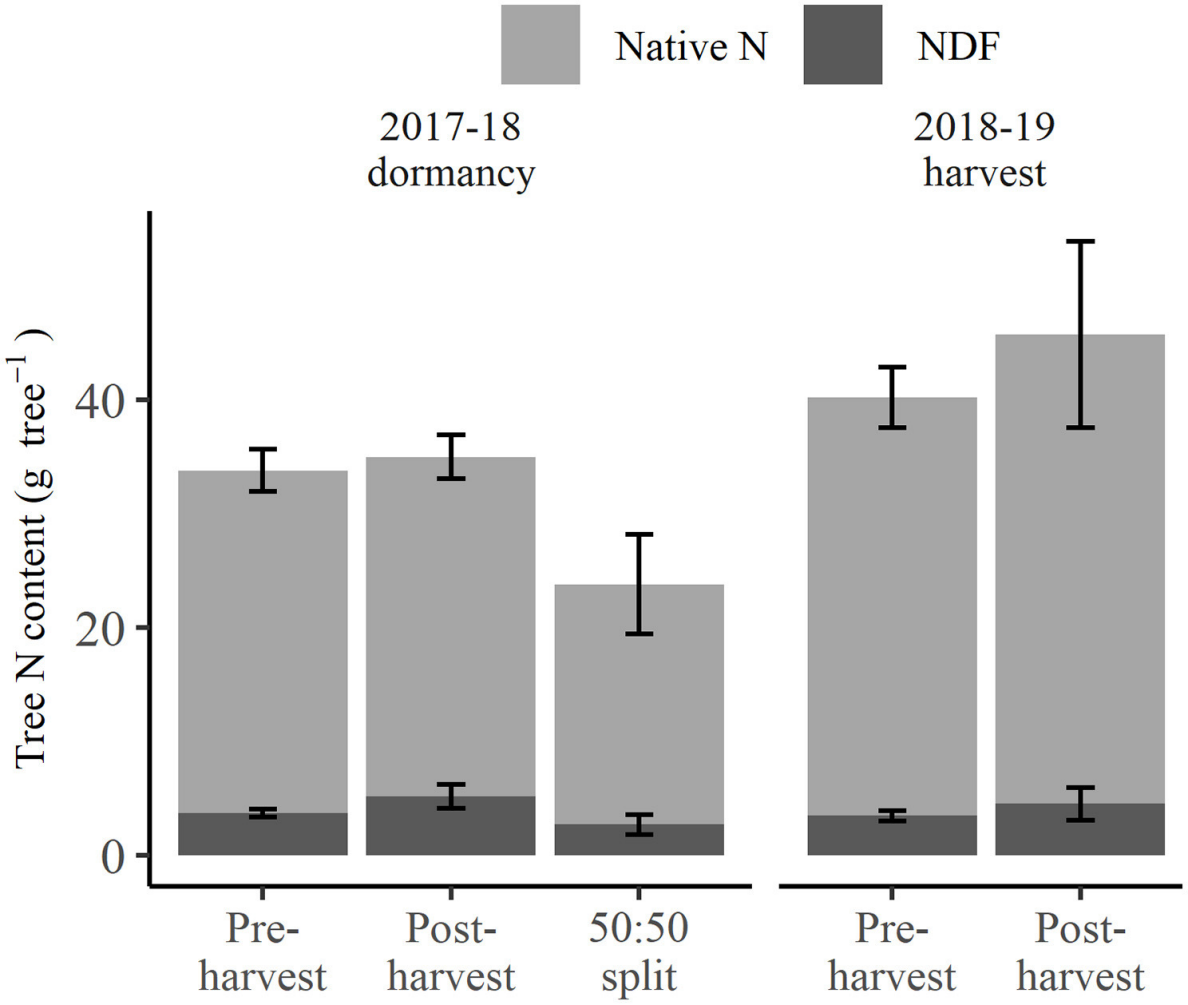
Amount of nitrogen derived from fertilizer (NDF) and unlabeled N (Native N) for N-treated trees destructively harvested at the 2018 dormancy (excluding fruit and leaf foliage) and the 2019 harvest (including fruit and leaf) under different N fertigation treatments. Error bars represent ±SEM, *n* = 4.

**Figure 3:**
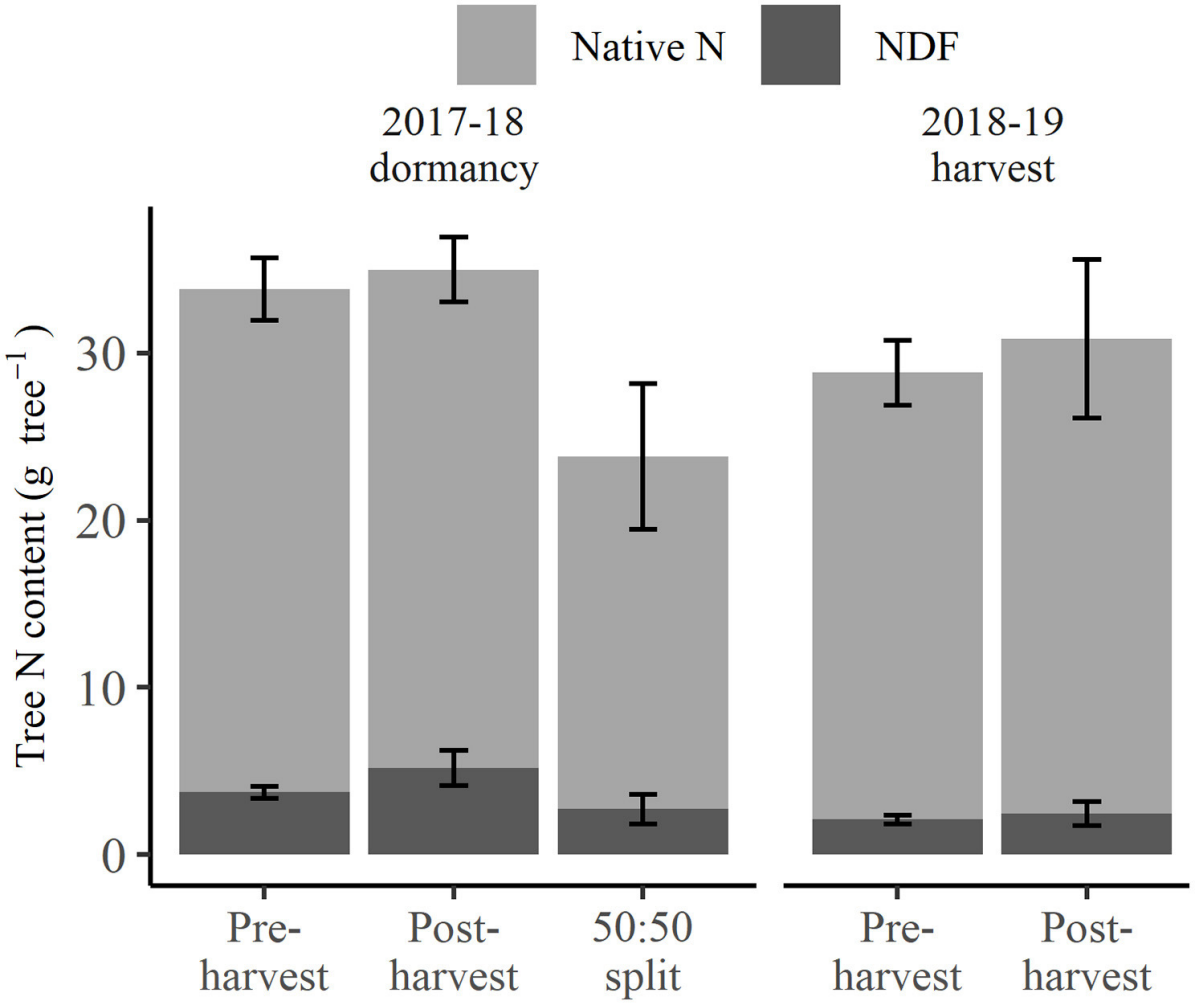
Amount of nitrogen derived from fertilizer (NDF) and unlabeled N (Native N) of perennial storage organs excluding fruit and leaf for N-treated trees destructively harvested at the 2018 dormancy and the 2019 harvest under different N fertigation treatments. Error bars represent ±SEM, *n* = 4.

At 2018 dormancy following treatment applications, trees that received pre-harvest and post-harvest treatments had an overall N status (Figure 2) of 33.8 and 35.0 g tree^−1^, respectively, not significantly different from each other, or the 23.8 g tree^−1^ for those of the 50:50 split treatment. At the same time, post-harvest treated trees showed a non-significant trend toward higher NDF (5.2 g tree^−1^) than pre-harvest or 50:50 split treated trees (3.7 and 2.7 g tree^−1^, respectively) (excluding fruit and leaf, Figure 2), yet their proportions of NDF to native N were not significantly different. At the 2019 harvest, the overall N status of trees that received the pre-harvest and post-harvest treatments, at 40.2 and 45.7 g tree^−1^, respectively (Figure 2), were neither significantly different and nor was their NDF status (3.5 and 4.5 g tree^−1^, respectively, Figure 2). Neither the native N nor NDF status of trees at 2018 dormancy significantly differed from those at the 2019 harvest, despite an apparent increase in native N status at the 2019 harvest. The exclusion of native N and NDF content of fruit and leaf organs represented the tree N storage status (Figure 3) at the 2019 harvest. Both tree native N and NDF storage status of any of the N-treated trees (Figure 3) did not significantly differ between 2018 dormancy and 2019 harvest.

From soil sampled at 0–10 cm depth at 2018 dormancy (July 11), the [N] was not significantly different between any treatments. In the same samples, [^15^N] of post-harvest and 50:50 split treatments were both significantly higher than that of the control treatment, whereas those from the pre-harvest treatment were not significant (Table 2).

**Table 2:** The [N] and [^15^N] of soil samples taken from 0 to 10 cm depth on July 11, 2018, during winter dormancy. Mean values ± SE, *n* = 4.

**Treatment**	**[N] (% dry weight)**	**[^**15**^N] (atom %)**
Control	0.143 ± 0.013	0.3696 ± 2.2 × 10^−4^
Pre-harvest	0.146 ± 0.008	0.3773 ± 2.0 × 10^−3^ ns
Post-harvest	0.139 ± 0.005	0.3811 ± 3.0 × 10^−3**^
50:50 split	0.152 ± 0.005	0.3873 ± 2.5 × 10^−3***^

[^15^N] values accompanied by a notation indicating the extent of difference from the Control for Dunnett's test: ns p > 0.05; **p < 0.01; ***p < 0.001.

### Leaf N Dynamics

**Figure 4:**
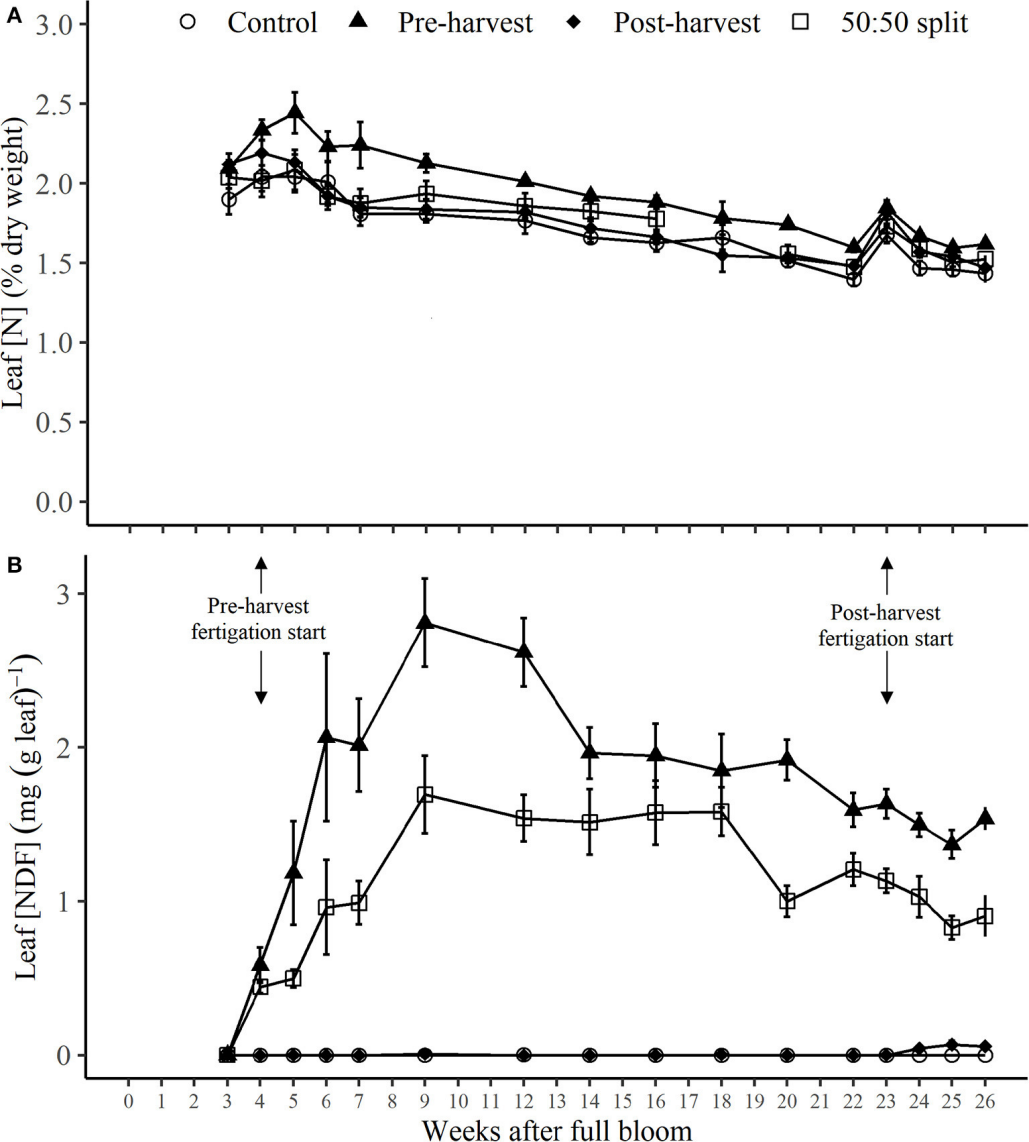
Dynamics of leaf N concentration [N] **(A)** and concentration of nitrogen derived from fertilizer (NDF) [NDF] **(B)** for N-treated trees during the 2017–2018 season following different N application timings. Error bars represent ±SEM, *n* = 4. Fruit was harvested at 22 WAFB.

Following the commencement of N application to pre-harvest and 50:50 split treatments at 4 WAFB, the leaf N concentration ([N], as % of leaf dry weight) of pre-harvest treated trees (Figure 4A) was significantly higher (*p* < 0.05) than that of post-harvest and control trees for most of the period 7–20 WAFB, after which differences between all treatments were not significant (see Supplementary Table 1). With a significant increase (*p* < 0.05) in leaf [N] of the pre-harvest trees (only) during their period of N application, all treatments were at maximum leaf [N] at or close to 5 WAFB, of 2.0 to 2.4%, followed by a generally slow decline throughout the remainder of the season to values of 1.4 to 1.6% at 26 WAFB (Figure 4A; Supplementary Table 1). There was one marked increase in leaf [N] of all treatments from 22 to 23 WAFB, following fruit harvest, but thereafter the decline in values resumed (Figure 4A; Supplementary Table 1).

Concentration of leaf NDF [(NDF), as mg NDF (g dry leaf) ^−1^] of pre-harvest and 50:50 split treated trees (Figure 4B) sharply increased after the first applications of N at 4 WAFB. From then, leaf [NDF] for pre-harvest and 50:50 treatments increased steadily to respective maxima of 2.8 and 1.7 mg N (g leaf) ^−1^ at 9 WAFB, before a gradual decrease to a respective 1.5 and 0.9 mg N (g leaf) ^−1^ at 26 WAFB (Figure 4B). Leaf [NDF] for the whole of the period 4–26 WAFB for pre-harvest treated trees and for 7–26 WAFB for the 50:50 split treatment (Figure 4B) was significantly higher (*p* < 0.05) than that of those treated post-harvest (commencing 23 WAFB). Also, for much of this latter period leaf [NDF] for the pre-harvest treatment was significantly higher (*p* < 0.05) than that of the 50:50 split treatment (Supplementary Table 1). The only reflection in leaf [NDF] of post-harvest N application was a slight, but insignificant, increase to a maximum of 0.07 mg N (g leaf) ^−1^ at 25 WAFB (Figure 4B; Supplementary Table 1).

### Nitrogen Allocation and Remobilization

**Figure 5:**
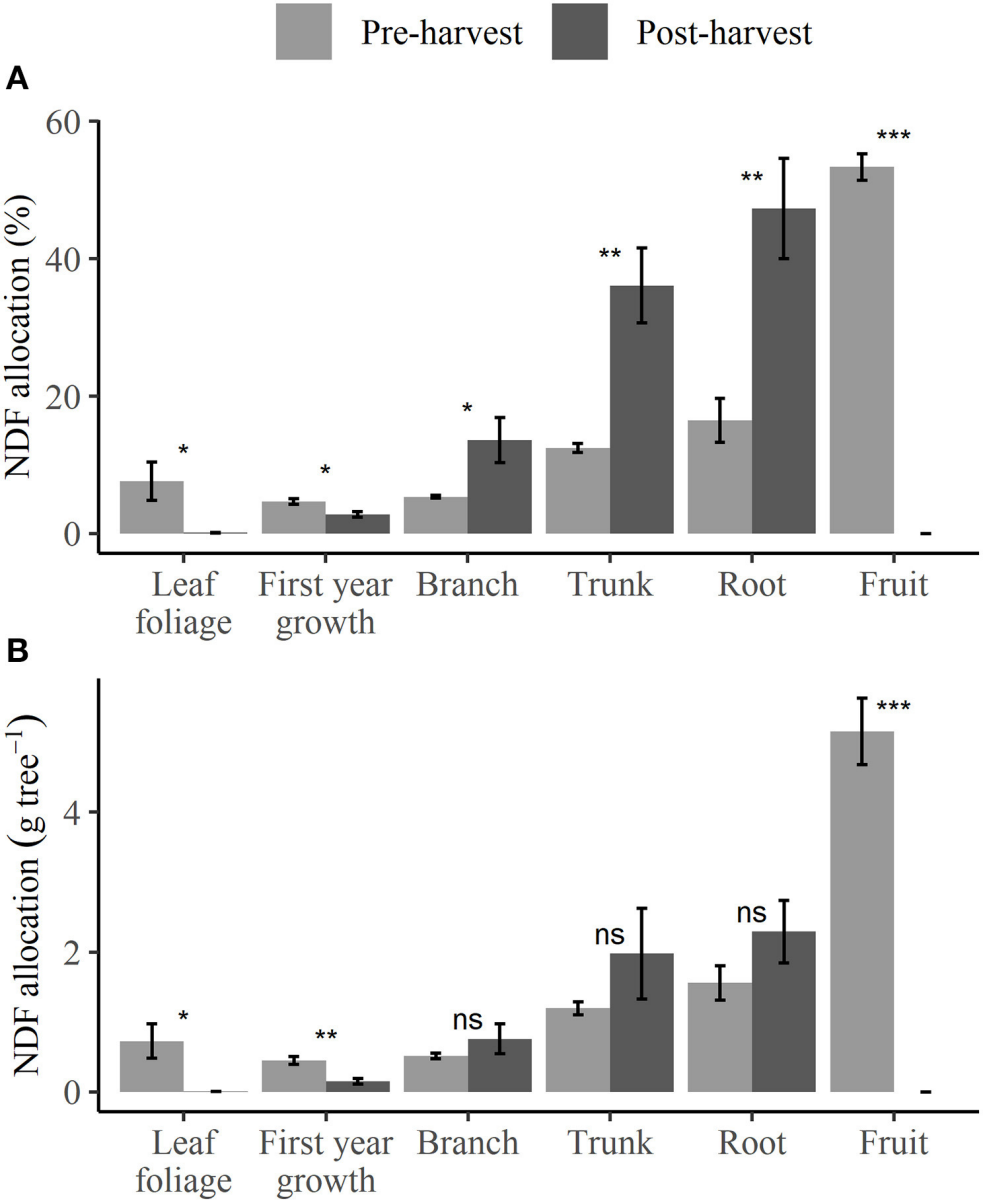
Proportion **(A)** and total amount **(B)** of nitrogen derived from fertilizer (NDF) allocated into different organs for N-treated trees destructively harvested at dormancy of the 2017–2018 season under different N application treatments. Error bars represent ±SEM, *n* = 4. For each organ, notation indicating the extent of difference between treatments from unpaired two-sample *t*-test: ns *p* > 0.05; *p < 0.05; **p < 0.01; ***p < 0.001.

The NDF_tree_ (including fruit) of pre-harvest treated trees was significantly higher (*p* < 0.05) and nearly double that of post-harvest and 50:50 split treated trees, at 9.6, 5.2, and 5.2 g N tree^−1^, respectively (Supplementary Table 2) at dormancy in 2018. Pre-harvest treated trees allocated significantly higher (*p* < 0.05) percentage of NDF into fruits, leaves, and other 1st-year growth organs (that included buds, spur growth, and 1st-year wood) of 53.0, 7.6, and 4.6%, respectively, than post-harvest treated trees of 0.0, 0.1, and 2.8%, respectively (Figure 5A). The amount of NDF allocated to fruits, leaves, and 1st-year growth organs (Figure 5B) were also significantly higher (*p* < 0.05) for pre-harvest treated trees than to those of post-harvest (Supplementary Table 2). In contrast, the percentage of NDF allocated to perennial organs (Figure 5A; branches, trunk, and roots) by post-harvest treated trees (13.6, 36.1, and 47.3%, respectively) was significantly higher (*p* < 0.05) than that of pre-harvest treated trees (5.4, 12.5, and 26%, respectively). However, despite the differences in the percentage of allocation, the amounts of NDF allocated to branches, trunk, or roots (Figure 5B) were not significantly different between pre- and post-harvest treatments.

**Figure 6:**
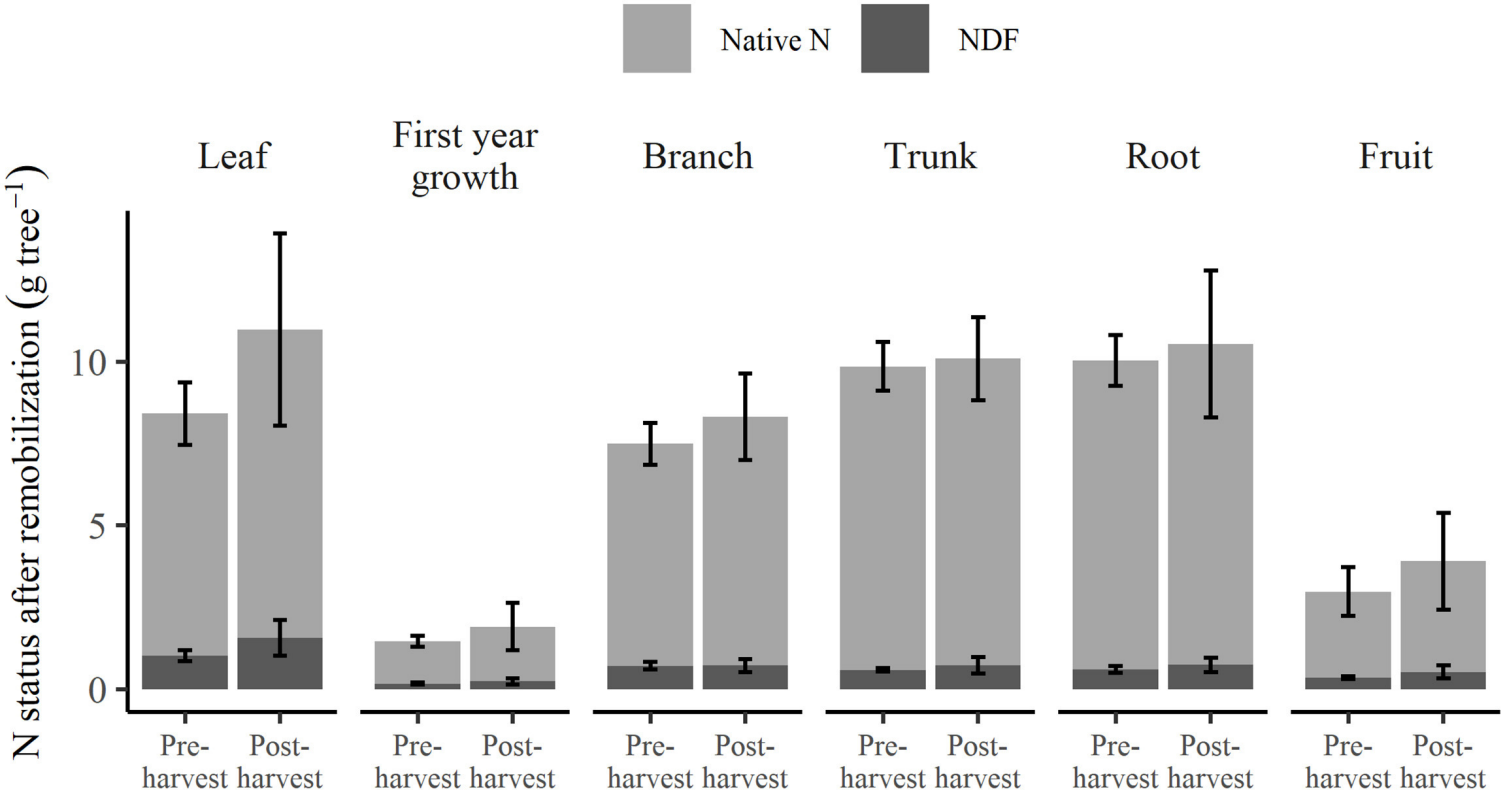
Total N in each organ showing composition of native N and nitrogen derived from fertilizer (NDF) at 1 week after fruit harvest of the 2018–2019 season under different N application treatments. Error bars represent ±SEM, *n* = 4.

The N status of each tree organ when the trees were destructively harvested in March 2019, 1 week after fruit harvest, is shown in Figure 6. The N status consisted of that remobilized in spring (native N and NDF stored from the previous season) and any native N and the remaining N_f_ taken up in the current season. For specific organs of these trees, no significant differences were found between pre- and post-harvest treatments, in their NDF or native N (Supplementary Tables 3, 5). For these pre- and post-harvest treatments, higher total N was observed in leaf (8.4 and 11.0 g tree^−1^, respectively) than in fruit (3.0 and 3.9 g tree^−1^).

**Figure 7:**
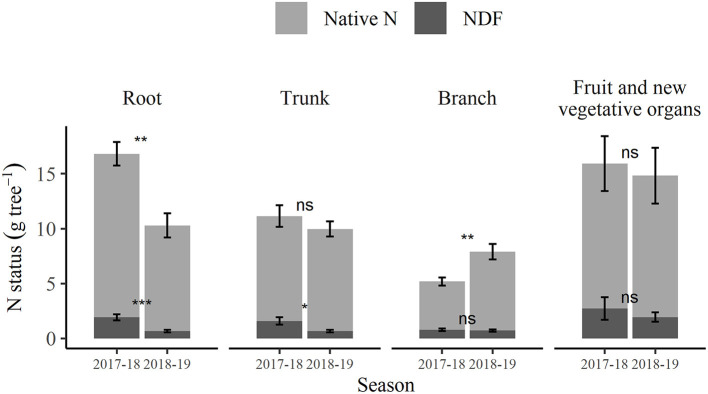
Total N contained in the roots, trunk, branches, and seasonal canopy showing the composition of N derived from native N and nitrogen derived from fertilizer (NDF) at winter dormancy of 2017–2018 season and at 1 week after fruit harvest timing of the 2018–2019 season (March 2019). Error bars represent ±SEM, *n* = 12. For each organ, notation indicating the extent of difference related to the time of sampling from unpaired two-sample *t*-test: ns *p* > 0.05; *p < 0.05; **p < 0.01; ***p < 0.001.

For either of the two seasons, the fertigation timing of pre- and post-harvest treatments did not result in significant differences in native N or NDF content within the tree organ groups of root, trunk, branch, and 1st-year wood, or in N content of bud and spur. Although there were significant differences in NDF content of both bud and spur between the pre- and post-harvest treatments at 2018 dormancy (only), the combined NDF total for these organs only constituted 6.7 and 1.0%, respectively, of their total tree NDF contents at that time. Hence, it was considered reasonable for the purpose of comparison of organ N and NDF contents between seasons to pool the two treatments (by taking the mean) to give season values for these two parameters of each organ group (Figure 7). The changes in N status of perennial organs from 2018 dormancy to 2019 harvest represent both the net native N and NDF remobilization and translocation, and any further N uptake from any source during that period. The total N status of root organs declined significantly (*p* < 0.05) between 2018 dormancy (15.2 g tree^−1^) and 2019 harvest (10.7 g tree^−1^) (Supplementary Tables 4, 5). Similarly, NDF in the roots and trunk declined significantly (*p* < 0.05) over the same period. However, the total N of the trunk (Figure 7) did not significantly change over that period. A comparison was made between the N and NDF content of branches, including first-year wood at 2018 dormancy with that of the same wood tissue following 2019 harvest, i.e., branches, not including new 1st-year growth. This comparison revealed that the total N (Supplementary Tables 4, 5) of branches increased significantly (*p* < 0.05) from 2018 dormancy (4.5 g tree^−1^) to 2019 harvest (7.9 g tree^−1^), whereas NDF (Supplementary Tables 2, 3) at 0.7 g N tree^−1^ was not significantly altered. Overall, a comparison of the (pooled) tree total N contents at 2018 dormancy with those following 2019 harvest found no significant changes in total N (34.41 and 42.99 g tree^−1^, respectively) or NDF (4.45 and 4.01 g tree^−1^, respectively); however, the tree total native N over the same period increased significantly (*p* < 0.05) from 30.65 to 38.97 g tree^−1^.

From the proportion perspective, NDF and native N stored in roots were 43.2 and 49.7%, respectively of their total tree contents at 2018 dormancy. The corresponding values for trunk and branch (including first-year wood) were 35.7 and 31.9% and 17.7 and 14.7%, respectively—in total, the NDF and native N in the three organs constituted >96% of their total tree content at 2018 dormancy. In each case of roots, trunk, and branch, the proportions of NDF and native N stored were not significantly different. At 2019 harvest, the proportions of NDF and native N stored in the branch (excluding 1st-year wood) were both 18.6% and were not significantly different. However, the corresponding values for trunk and root were 15.7 and 24.5% and 17.2 and 24.7%, respectively and for each the proportions of NDF and native N were significantly different (*p* <0.05). In total, the three organs constituted 51.5 and 67.8% of the tree total NDF and native N contents at the 2019 harvest. The remaining 48.5 and 32.2% of tree total NDF and native N contributed to the new growth of fruit and vegetative organs.

### Fruit Quality

Fruit yield and quality attributes (Tables 3A,B), except for the dry matter content and skin color, were not significantly different between treatments within each season of assessment. In the first season, post-harvest treated trees had fruit of significantly higher (*p* < 0.05) dry matter content than that of 50:50 split treated trees. Fruit of the post-harvest treatment also had significantly higher (*p* < 0.05) a^*^ index (higher a^*^ indicates more redness), higher peel redness, and red color coverage than that of the 50:50 split treatment in the first season. Also in the first season, the fruit of the post-harvest trees had a significantly yellower (*p* < 0.05) peel background color than that of the 50:50 split treatment.

**Table 3A:** Fruit yield and quality parameters at fruit harvest and post-harvest in 2017–2018 season under the impact of different N fertigation timings. Within each parameter, different letters indicate significant differences (*p* < 0.05) between treatment means (*n* = 4).

**Treatment**	**Yield (kg tree^−1^)**	**Weight (g)**	**Dry matter content (%)**	**L index**	**a index**	**Delta absorbance**	**Red intensity**	**Red coverage**
Control	16.98	166.22	13.45 ab	44.11 ab	32.07 a	0.33	5.50 ab	4.03 a
Pre-harvest	18.42	155.95	13.31 ab	43.71 ab	31.91 ab	0.40	5.69 ab	3.99 ab
Post-harvest	14.57	172.59	14.19 a	42.16 b	32.78 a	0.33	6.43 a	4.38 a
50:50 split	14.63	138.12	12.43 b	47.65 a	27.71 b	0.47	4.30 b	3.28 b
	**Background color**	**Length (mm)**	**Diameter (mm)**	**Firmness (kg)**	**Starch index**	**TSS (Brix°)**	**Post-harvest firmness (kg)**	**Post-harvest TSS (Brix°)**
Control	7.73 ab	69.71	70.65	7.61	5.46	11.80	5.69	11.70
Pre-harvest	7.62 ab	68.07	69.67	8.11	5.68	11.45	5.38	11.55
Post-harvest	8.09 a	70.64	71.68	7.56	5.15	12.21	5.81	12.16
50:50 split	6.64 b	64.99	66.52	7.89	5.52	10.78	5.59	11.02

*°Represents degree of and angle, the parameter was measured in “Brix degree angle”*.

**Table 3B:** Fruit yield and quality parameters at fruit harvest and post-harvest in 2018–2019 season under the impact of different N fertigation timings. Within each parameter, different letters indicate significant differences (*p* < 0.05) between treatment means (*n* = 4).

**Treatment**	**Yield (kg tree^−1^)**	**Weight (g)**	**Dry matter content (%)**	**L index**	**a index**	**Delta absorbance**	**Red intensity**	**Red coverage**
Control	6.84	156.71	15.18	45.74	33.06	0.45	5.50	3.83
Pre-harvest	8.1	176.48	14.64	43.32	35.89	0.45	5.96	4.31
Post-harvest	9.56	173.79	14.94	44.98	34.43	0.45	5.32	3.79
	**Background color**	**Length (mm)**	**Diameter (mm)**	**Firmness (kg)**	**Starch index**	**TSS (Brix°)**	**Post-harvest firmness (kg)**	**Post-harvest TSS (Brix°)**
Control	6.67	71.69	66.55	9.14	4.87	14.17	6.45	14.05
Pre-harvest	6.74	75.17	68.98	8.82	4.68	13.58	6.49	14.33
Post-harvest	6.95	73.54	69.06	8.91	4.98	14.08	6.64	14.20

*°Represents degree of and angle, the parameter was measured in “Brix degree angle”*.

## Discussion

In an intensive apple orchard, this study found that the uptake of fertilizer N was most efficient when sink organs were actively growing in spring, and mature trees relied on the remobilization of stored N for new growth that can buffer single-season variation in N supply. When N was applied post-harvest, the allocation of NDF to tree organs was predominantly to storage organs with little allocation to leaves (Figure 4). In contrast, leaf [NDF] was found to be a good indicator for the timing of uptake of N-applied pre-harvest treatment (Figure 4B, pre-harvest and 50:50 split treatments), and greater NDF was allocated to spurs and buds in the pre-harvest applied N treatment. These differences and their importance in the timing and uptake of applied N, its allocation to tree organs, and its remobilization for the following season are examined in detail in the discussion that follows.

### Nitrogen Uptake and Allocation

Pre-harvest N application had NUpE of 32.0% in the 2017–2018 season, nearly double that of the other treatments (Figure 1; ~17.2%). Although the 50:50 split treatment received (up to harvest) only half the rate of N of the pre-harvest treatment, its NUpE was not significantly different from that of the post-harvest treatment. This suggests that NUpE was dependent on the availability of N_f_ in the soil at the time of highest demand and thus, a crucial factor in optimizing NUpE. Comparable NUpE was found for 3-year-old apple trees (22.3%, Neilsen et al., 2001b) and apple trees in newly planted orchards (16–19%, Neilsen et al., 2001a) with pre-harvest N applied *via* dripper fertigation. San-Martino et al. (2010) also found that NUpE was higher with spring N fertilizer application (65.7%), compared to summer application (37.4%) in 7-year-old sweet cherry trees. The much higher NUpE of that study relative to the current study might be due to a combination of: hand application of fertilizer below the soil surface; installation of plastic barriers 2 m from the trunk and 1 m deep around each tree; and the application of ammonium nitrate, with ammonium being much less prone to leaching than nitrate—these factors all potentially reducing N loss (San-Martino et al., 2010). In addition, the extended post-harvest transpiration of cherry as a summer crop, relative to that of apple with a later harvest, could account for greater N uptake in the intervening period. The latter factor is also consistent with the much-reduced NUpE of post-harvest fertilizer application observed in our study, where the amount of N taken up is likely to be limited by the lessened transpirational pull associated with the reduced sap flow activity (Fujii and Kennedy, 1985) later in the season. Although there were indications of some fertilizer N remaining in the soil at 2018 dormancy (Table 2), it is very likely that some of that not utilized by the trees may have been lost to the immediate environment either *via* leaching (Hardie et al., 2018) taken up by roots of neighboring trees or undergone a transformation to other N forms, e.g., by denitrification to nitrous oxide (Swarts et al., 2016), dinitrogen gases, or by dissimilatory nitrate reduction to ammonium (Giblin et al., 2013).

A significantly higher (*p* < 0.05) proportion of NDF was allocated to perennial organs from post-harvest applied N than from the pre-harvest or 50:50 split treatments (Supplementary Table 2). However, given the reduced NUpE of post-harvest applied N, the absolute amount (as opposed to %) of NDF allocated into perennial organs was not significantly different between treatments (Figure 5B; Supplementary Table 2). This indicates that the allocation of NDF was dependent on the timing of N application, as has been reported in sweet cherry (San-Martino et al., 2010) and nectarine (Tagliavini et al., 1999). A large difference in the amount of NDF allocated to leaves, buds, and spurs was observed between treatments. Post-harvest treated trees allocated 0.01 and 0.04 g NDF tree^−1^ into buds and spurs, respectively, each significantly less (*p* < 0.05) than the respective 0.04 and 0.21 g NDF tree^−1^ for pre-harvest treated trees (Supplementary Table 2). This suggests that the allocation of N into the buds and spurs occurred earlier in the season rather than post-harvest and so can depend on early season soil N availability. Strong additional support for this proposal is found in the % of N in the buds and spurs that were derived from fertilizer (Supplementary Table 2), in each case there being significant declines (*p* < 0.05) from pre-harvest > 50:50 split > post-harvest. Furthermore, it has been shown that for apple trees, subsequent season buds develop in early summer (Landsberg, 1974), and this is likely to be improved by non-limiting N supply during that period. These results contradict the general grower practice of applying N post-harvest with the intent to improve N nutrition of buds and spurs, and subsequently improving fruit quality in the following season (Rainham, 2016).

### Influence of N Application Timing on Leaf N Dynamics

The rapid uptake of applied N by the pre-harvest treated trees (Figure 4A) was reflected in their leaf [N] becoming significantly higher (*p* < 0.05) than for the post-harvest and control treatments from 3 weeks after the commencement of the pre-harvest N application. This remained so, with little exception, until fruit harvest at 22 WAFB (Supplementary Table 1). The leaf [N] for the 50:50 split treatment did not differ significantly from any other treatment throughout the monitored period (except at 20 WAFB, this being of seemingly little consequence). The rapid uptake of applied N by pre-harvest and 50:50 split treated trees was also clear from their marked increase in leaf [NDF], following the commencement of N application (Figure 4B). Leaf [N] of all treatments peaked at around 4–5 WAFB (1 week after pre-harvest N application commenced), which coincided with the commencement of fruit cell expansion. From that time, leaf [N] decreased gradually over the season in all treatments until fruit harvest, as it has been reported elsewhere (Aguirre et al., 2001; Grassi et al., 2002). The gradual seasonal decrease is likely due to the fruit becoming a stronger sink for N than leaves as the season progresses (Neilsen et al., 2005), with the result of a decline in the ratio of N content to leaf biomass.

Leaf [N] increased sharply a week after the fruit harvest at 22 WAFB for all treatments (Figure 4A). This rapid increase indicates that prior to the harvest; the fruit was a stronger sink for N than leaves and that with the fruit removed, N from ongoing uptake and/or internal re-allocation led, *via* vascular transfer, to a brief surge in leaf [N]. From this point, onward leaf [N] continued to drop, this being consistent with the onset of leaf senescence accompanied by a reduction of chlorophyll level and related activity (Fang et al., 1998), even when N availability was high as with the post-harvest application (Figure 4B). In addition, the sink of N became oriented toward storage organs in the lead-up to dormancy (Munoz et al., 1993). Thus, the reduction in transpiration-facilitated N uptake in combination with N translocation to perennial organs can explain the reduced N demand and uptake of post-harvest treated trees.

The dynamics of leaf [NDF] clearly reflected the movement of all pre-harvest NDF into or away from the leaf after N application (Figure 4B). After the pre-harvest N application commenced at 4 WAFB, leaf [NDF] increased rapidly until 9 WAFB (1 week after cessation of all pre-harvest N application), before decreasing slowly toward dormancy. Over the period 4–9 WAFB, the rate of NDF uptake for the pre-harvest treatment was almost double that of the 50:50 split treatment, indicative of its greater N supply. At the commencement of leaf fall (26 WAFB), leaf [NDF] had decreased to 1.7 and 0.9 mg NDF (g leaf) ^−1^ for pre-harvest and 50:50 split treated trees, respectively, from their respective 9 WAFB maxima of 2.8 and 1.7 mg NDF (g leaf) ^−1^. This decline indicated that a respective 39% and 47% of NDF was either translocated to fruit or recycled into perennial organs before dormancy. The proportions of NDF from leaves translocated into fruit or withdrawn into storage could not be distinguished, as the labeled N in both fruit and perennial organs could have originated from either root N uptake or translocation. Millard and Thomson (1989) reported that 1-year-old apple trees that were fertigated with N withdrew ~69% (summer fertigation) or 41% (autumn fertigation) of N from the leaves into perennial storage organs prior to dormancy. Although the N withdrawal from that summer fertigation (Millard and Thomson, 1989) was 30% higher than of the pre-harvest treatment of our finding, these trees did not have fruit; thus, the impact of crop load on N withdrawal to perennial organs cannot be determined. Importantly, leaf [NDF] of our post-harvest treatment was 0.0 mg NDF (g leaf) ^−1^ for the duration of the experiment (Figure 4B), with only an insignificant increase from 24 to 26 WAFB, indicating a little relationship between N supplied and leaf [NDF] after the fruit harvest. The poor relationship between fertilizer N supply and leaf [NDF] for the post-harvest treatment, in addition to the preferred allocation of post-harvest NDF toward perennial organs, gives further support that the tree has greatly reduced N demand post-harvest, at a time when it has begun to shift physiologically from an active, growing phase toward a winter, dormant phase (Fadón et al., 2020).

### Nitrogen Storage and Remobilization

Tree N status at 2018 dormancy was comprised of NDF and native N of the tree organs of roots, trunk, branches, and 1st-year wood. By destructive harvest in 2019, the NDF components of these treatments, including that in removed fruit and leaf, showed no significant change and in fact, a slight decrease (Figure 2). In contrast, there were indications (inconclusive) of continued uptake of native N (Figure 2). This outcome strongly suggests that despite there being elevated ^15^N content in the soil sampled from the post-harvest and 50:50 split treatments at 2018 dormancy (Table 2), there was no significant uptake from the soil of any remaining N_f_ between that time and the destructive harvest in 2019. Consequently, we consider that the NDF component of the 2019 harvested trees, including that removed in fruit and leaf, to have come solely from remobilization of that stored at 2018 dormancy. On the other hand, the native N component of the trees was made up of remobilized N, and possibly an additional amount is taken up from the soil following the 2018 dormancy, with it not being possible to apportion their relative contributions.

Although the NDF taken up by pre-harvest treated trees (9.6 g NDF tree^−1^) was significantly more (*p* < 0.05) than that of the other applied N treatments (5.2 g NDF tree^−1^), the difference of 4.4 g NDF tree^−1^ was only equivalent to ~10% of tree N status at 2018 dormancy, at which time the overall tree N status (Figure 2) did not significantly differ between any applied N treatments. This was because: (1) NDF made up only a small portion of N status of the trees, and (2) 60% and 47% of NDF taken up by pre-harvest and 50:50 split treated trees, respectively, was allocated to fruit and leaves and lost from the system *via* fruit harvest and leaf fall (Supplementary Table 2). On the other hand, at 2019 harvest, both tree native N and NDF storage status (fruit and leaf organs excluded, Figure 3) of pre- and post-harvest treated trees were not significantly different from those of the previous season when no additional N had been provided in the interim. This suggests that the 14-year-old trees had an N storage capacity sufficient to buffer the impact of a single season variation in N supply. In contrast, it has been demonstrated that levels of remobilized N in much younger trees with less biomass of perennial organs (and presumably less N storage capacity) were highly dependent on the rate of N supplied the previous season (Millard and Proe, 1993; Cheng and Fuchigami, 2002). Hence, while N status at dormancy is particularly important for deciduous fruit trees, as it determines the N available for the remobilization in the following season, it is very likely that the age/size of a tree is an influence in its ability to buffer seasonal variation in soil N availability related to environmental conditions or orchard practices.

Remobilization of stored N into fruit and new vegetative organs has been found to be critical to fruit development (Neilsen et al., 2001a) and shoot growth (Millard et al., 2006). From this study, despite the significantly higher (*p* < 0.05) amounts of NDF stored in both buds and spurs of the pre- than post-harvest treated trees at 2018 dormancy (Supplementary Table 2), the amounts of NDF remobilized into fruit, leaves, and 1st-year growth organs in the 2018–2019 season (Figure 6) did not significantly differ between the treatments. This indicates that the amount of NDF remobilized into new growth was not affected by the differences in NDF of bud and spur organs. This is perhaps to be expected, as the N content in buds and spurs only contributed ~3% of the N content of the whole trees, and of that 3%, ~87% was constituted of native N. A study of 3-year-old apple trees compared low (30 mg dm^−3^) and high (150 mg dm^−3^) N supply, fertigated in summer and found that remobilized N contributed a respective 87 and 61% of total N to shoot tissues (spur, shoot, and reproductive tissues; Guak et al., 2003). Another study of 2-year-old apple trees found that the contribution of remobilized N in stems to leaf growth was 28 and 34%, respectively when N was supplied in the spring or autumn of the previous season (Millard and Thomson, 1989). The findings from these two trials suggest that stored N in older trees generally provides a greater proportion of N requirements of the following season than in younger trees. We found strong evidence for this in our 14-year-old apple trees where 48.5% (pooled data) of stored NDF from 2017 to 2018 season was remobilized to newly grown tissues in the subsequent season and, this was only 13.3% of total N in new growth (Figure 7). This implies that, with there not being a significant increase in total N content of the trees between seasons, the 87% of total N required for new growth came from the previous seasons' reserves. A review of other studies has also concluded that the contribution from previously-stored N relative to NDF stored from the previous single-season may generally increase with tree age (Millard and Grelet, 2010).

Our finding that N remobilization was not affected by the timing of N application was not consistent with the report of preferential remobilization of N taken up in autumn over that taken up in spring and summer by 2-year-old apple trees (Millard and Thomson, 1989). Again, the substantially greater N reserves likely to have been held within our 14-year-old trees quite possibly played a part in the insignificant impact of N application timing on N remobilization found in our study, as NDF contributed only a small fraction (9.1%) of total remobilized N. If ^15^N-labeled N had been applied to our older trees at different timings for multiple seasons (e.g., ≥ 2), any significant impact on remobilization of N related to application timing might have been more clearly identified.

Between 2018 dormancy and 2019 harvest, significantly decreased (*p* < 0.05) native N and NDF were found in the roots and NDF only in the trunk. Decreased N in the roots and trunk coincided with elevated N levels in fruit and new vegetative growth. A significant increase (*p* < 0.05) of native N in branches was also observed during that period. However, the substantial amount of native N within the trunk did not vary between seasons. These changes, between 2018 dormancy and 2019 harvest, suggest that roots were the main source of both remobilized NDF and native N. The trunk was also another main source of NDF and the main sinks in the 2019 harvest, for both were new vegetative growth and fruit and; branches for native N only. It is important to acknowledge that the inter-seasonal increase of native N in branches, vegetative organs, and fruit was 11.0 g N tree^−1^ greater than its decrease from the root organ. This suggests that the increase in native N in branches, vegetative organs, and fruit could originate from native N remobilized from roots and in addition, some soil N uptake throughout the 2018–2019 season. Further native N uptake is supported by the significant increase (*p* < 0.05) of 8.3 g N tree^−1^ in native N status found at the 2019 harvest, despite there being no significant change in NDF, with this increase not being significantly different from the aforementioned 11.0 g N tree^−1^.

Studies of 2-year-old trees found that apples utilized the main trunk (Millard and Neilsen, 1989) and sweet cherry utilized roots (Grassi et al., 2002) as their primary sources of remobilized N. However, a comparison of non-fruiting young trees of these studies with the remobilization characteristics of the 14-year-old trees of our study would seem difficult. We conclude that for older trees, with relatively high capacity for N storage, the effects of seasonal N deficit are buffered. This is well-supported by our findings that withholding of additional N supply to the 14-year-old trees for one season had no significant impact on fruit yield, quality, or growth of the new season. Such an N buffering capacity of mature deciduous trees could explain why the impact of many N fertilization studies requires multiple seasons of repeated treatments to show a significant impact on fruit quality. Indeed, in this study, there were limited differences in fruit quality outcomes associated with the timing of N application. Importantly, this indicates that pre-harvest N taken up into fruit does not negatively impact color development or firmness, as this has been reported in some cases where excessive N was applied pre-harvest (Shear and Faust, 1980; Neilsen et al., 1999).

## Conclusions

From our application of ^15^N-enriched fertilizer to commercial apple trees at different timings, we have made important findings that could assist industry in the better utilization of N to the benefit of growers and the environment. We found that the significantly highest NUpE (32%) of the pre-harvest treated trees when compared with that of the post-harvest and 50:50 split treatments (each ~17.2%), was likely due to soil N availability being greatest during the period of highest tree N demand. Such demand was likely driven by fruit development in combination with canopy and new wood tissue development occurring predominately in early to mid-season. In contrast, the lower NUpE by the post-harvest treated trees was likely related to the reduced N demand as the physiology of the fruit-free trees shifted toward dormancy. Our results clearly indicate that pre-harvest N application optimizes fertilizer N uptake, with no impact on fruit yield or quality or compromise to winter N storage. Additionally, this practice would leave less fertilizer N in the soil with the potential to cause environmental pollution. Thus, the commencement of supply of pre-harvest N fertilizer at around 4 weeks after full bloom can be recommended. The uptake of such supply can be clearly traced by the leaf N analysis, unlike that of post-harvest N uptake where such analysis, as a result of contrasting N allocation, is of little benefit.

Growers commonly practice post-harvest N application with the aim of increasing stored N in buds, spurs, and roots to be remobilized in the following season for improved bloom and fruit quality. However, our results indicate greater uptake into buds and spurs from pre-harvest N application, and the practice of post-harvest application of N for storage to support the growth of the next season is neither recommended from a resource use efficiency perspective nor as a practice to improve fruit quality. This study, through considerably improved understanding of N dynamics in mature apple trees, has important implications for commercial producers by indicating that N fertilizer strategies need to pivot from post-harvest to primarily pre-harvest N application. Such a change will assist in reducing N lost to the environment while providing adequate N supply to meet tree demands.

## Data Availability Statement

The original contributions presented in the study are included in the article/Supplementary Material, further inquiries can be directed to the corresponding author/s.

## Author Contributions

NS and DC conceptualized the study. BT, NS, and DC designed the experiment. BT, NS, and PQ implemented the methodology. BT and NS executed the experiment and data collection. BT and PQ performed the statistical analysis and summarized results in consultation with NS and DC. BT took the lead in writing the manuscript, received critical feedback, and edits from all co-authors. All authors discussed the interpretation of the data and results.

## Conflict of Interest

The authors declare that the research was conducted in the absence of any commercial or financial relationships that could be construed as a potential conflict of interest.

## Abbreviations

NDF: nitrogen derived from fertilizer
NUpE: nitrogen uptake efficiency
NRec: nitrogen recovery efficiency
TSS: total soluble solids
WAFB: weeks after full bloom.
