# Supplementary material for: Nitrogen Use Efficiency, Allocation, and Remobilization in Apple Trees: Uptake Is Optimized With Pre-harvest N Supply
Source: Front Plant Sci. 2021 May 31;12:657070. doi: 10.3389/fpls.2021.657070 (PMC8202005; doi:10.3389/fpls.2021.657070)
Supplement: Supplementary file 1 [file Data_Sheet_1.docx]

Supplementary Material

## Supplementary Tables

**Supplementary Table 1** Nitrogen concentration, and N derived from fertilizer of leaf samples collected over the 2017-18 season under the impact of different N fertigation timings. Within each sampling week, different letters indicate significant differences (*p* < 0.05) between treatment means within that parameter (n = 4).

| **Week after full bloom** | **3** | **4** | **5** | **6** | **7** | **9** | **12** | **14** | **16** | **18** | **20** | **22** | **23** | **24** | **25** | **26** |
| --- | --- | --- | --- | --- | --- | --- | --- | --- | --- | --- | --- | --- | --- | --- | --- | --- |
| **Treatment** | **N concentration (% dry weight)** | | | | | | | | | | | | | | | |
| Control | 1.9 | 2.04 | 2.04 b | 2.01 | 1.81 b | 1.81 b | 1.77 b | 1.66 b | 1.63 b | 1.66 | 1.52 b | 1.40 b | 1.68 | 1.47 b | 1.46 b | 1.44 b |
| Pre-harvest | 2.1 | 2.34 | 2.44 a | 2.23 | 2.24 a | 2.13 a | 2.01 a | 1.92 a | 1.88 a | 1.78 | 1.74 a | 1.60 a | 1.85 | 1.67 a | 1.60 a | 1.62 a |
| Post-harvest | 2.12 | 2.19 | 2.13 ab | 1.92 | 1.85 b | 1.84 b | 1.82 ab | 1.72 b | 1.66 b | 1.55 | 1.53 b | 1.48 ab | 1.82 | 1.57 ab | 1.54 ab | 1.47 b |
| 50:50 split | 2.04 | 2.02 | 2.09 ab | 1.92 | 1.88 ab | 1.94 ab | 1.86 ab | 1.83 ab | 1.78 ab | 1.94 | 1.56 b | 1.48 ab | 1.73 | 1.59 ab | 1.50 ab | 1.53 ab |
|  | **N derived from fertilizer (mg N (g leaf)^-1^)** | | | | | | | | | | | | | | | |
| Control | 0 | 0.00 b | 0.00 b | 0.00 | 0.00 c | 0.00 c | 0.00 c | 0.00 b | 0.00 b | 0.00 b | 0.00 c | 0.00 c | 0.00 c | 0.00 c | 0.00 c | 0.00 c |
| Pre-harvest | 0 | 0.58 a | 1.18 a | 2.07 a | 2.02 a | 2.81 a | 2.62 a | 1.96 a | 1.95 a | 1.85 a | 1.92 a | 1.59 a | 1.63 a | 1.50 a | 1.37 a | 1.54 a |
| Post-harvest | 0 | 0.00 b | 0.00 b | 0.00 b | 0.00 c | 0.00 c | 0.00 c | 0.00 b | 0.00 b | 0.00 b | 0.00 c | 0.00 c | 0.00 c | 0.04 c | 0.07 c | 0.06 c |
| 50:50 split | 0 | 0.44 a | 0.50 ab | 0.96 ab | 0.99 b | 1.70 b | 1.54 b | 1.51 a | 1.58 a | 1.58 a | 1.00 b | 1.21 b | 1.13 b | 1.03 b | 0.83 b | 0.90 b |
|  | **^15^N atom percentage (%)** | | | | | | | | | | | | | | | |
| Control | 0.3689 | 0.3670 b | 0.3652 b | 0.3641 b | 0.3631 c | 0.3719 c | 0.3744 c | 0.3741 b | 0.3756 b | 0.3696 b | 0.3697 c | 0.3695 b | 0.3703 c | 0.3695 c | 0.3689 c | 0.3701 c |
| Pre-harvest | 0.3692 | 0.4912 a | 0.6039 a | 0.8023 a | 0.8148 a | 1.0149 a | 1.0110 a | 0.8760 a | 0.8759 a | 1.3205 a | 0.9148 a | 0.8600 a | 0.8060 a | 0.8131 a | 0.7932 a | 0.8385 a |
| Post-harvest | 0.3688 | 0.3671 b | 0.3653 b | 0.3645 b | 0.3629 c | 0.3710 c | 0.3746 c | 0.3742 b | 0.3748 b | 0.3698 b | 0.3697 c | 0.3694 b | 0.3749 c | 0.3824 c | 0.3902 c | 0.3881 c |
| 50:50 split | 0.3689 | 0.4772 a | 0.4866 ab | 0.6102 ab | 0.6291 b | 0.8034 b | 0.7782 b | 0.7759 a | 0.8039 a | 1.1609 a | 0.6844 b | 0.7723 a | 0.6912 b | 0.6869 b | 0.6401 b | 0.6598 b |

**Supplementary Table 2** Nitrogen derived from fertilizer (NDF), total NDF and NDF allocated to separate tree organs at dormancy in June 2018, under the impact of different N application timings. For specific organs, different letters indicate significant differences (*p* < 0.05) between treatment means (n = 4).

| **Organ type** | **Bud** | **Spur** | **Leaf foliage** | **First-year wood** | **Branch** | **Trunk** | **Old root** | **Fine root** | **Fruit** |  |
| --- | --- | --- | --- | --- | --- | --- | --- | --- | --- | --- |
| **Treatment** | **N derived from fertilizer (%)** | | | | | | | | | |
| Control | 0.00 d | 0.00 c | 0.00 c | 0.00 c | 0.00 b | 0.00 b | 0.00 b | 0.00 c | 0.00 b |  |
| Pre-harvest | 22.70 a | 18.87 a | 12.69 a | 25.48 a | 14.09 a | 11.39 a | 26.61 a | 6.17 b | 26.31 a |  |
| Post-harvest | 6.38 c | 3.82 c | 0.22 c | 11.90 b | 15.03 a | 16.00 a | 41.91 a | 12.66 a | 0.00 b |  |
| 50:50 split | 15.01 b | 13.18 b | 7.48 b | 16.62 b | 10.02 a | 11.31 a | 32.14 a | 9.31 ab | 19.27 a |  |
|  | **Total NDF (g tree^-1^)** | | | | | | | | | |
| Control | 0.00 c | 0.00 c | 0.00 b | 0.00 b | 0.00 b | 0.00 b | 0.00 b | 0.00 b | 0.00 c |  |
| Pre-harvest | 0.04 a | 0.21 a | 0.73 a | 0.20 a | 0.52 a | 1.20 ab | 1.46 a | 0.10 a | 5.15 a |  |
| Post-harvest | 0.01 bc | 0.04 c | 0.01 b | 0.10 ab | 0.76 a | 1.98 a | 2.10 a | 0.19 a | 0.00 c |  |
| 50:50 split | 0.02 ab | 0.13 b | 0.29 ab | 0.06 b | 0.28 ab | 0.84 ab | 1.30 ab | 0.10 a | 2.14 b |  |
|  | **NDF allocation (%)** | | | | | | | | | |
| Control | 0.00 c | 0.00 c | 0.00 b | 0.00 b | 0.00 b | 0.00 c | 0.00 c | 0.00 b | 0.00 b |  |
| Pre-harvest | 0.38 ab | 2.23 a | 7.64 a | 2.07 a | 5.37 b | 12.48 bc | 15.43 b | 1.05 ab | 53.36 a |  |
| Post-harvest | 0.21 b | 0.64 b | 0.14 b | 1.94 a | 13.64 a | 36.11 a | 42.53 a | 4.78 a | 0.00 b |  |
| 50:50 split | 0.48 a | 2.41 a | 5.97 ab | 1.16 ab | 5.06 b | 14.24 b | 24.18 b | 1.85 ab | 44.65 a |  |

**Supplementary Table 3** Nitrogen derived from fertilizer (NDF) percentage, total NDF and NDF allocation of various tree organs harvested at fruit harvest in March 2019 under the impact of different N treatment timings. For specific organs, different letters indicate significant differences (*p* < 0.05) between treatment means (n = 4).

| **Organ type** | **Bud** | **Spur** | **Leaf** | **First-year wood** | **Branch** | **Trunk** | **Old root** | **Fine root** | **Fruit** |  |
| --- | --- | --- | --- | --- | --- | --- | --- | --- | --- | --- |
| **Treatment** | **N derived from fertilizer (%)** | | | | | | | | | |
| Control | 0.00 b | 0.00 b | 0.00 b | 0.00 b | 0.00 b | 0.00 b | 0.00 b | 0.00 b | 0.00 b |  |
| Pre-harvest | 10.63 a | 11.73 a | 12.11 a | 11.94 a | 9.47 a | 18.06 a | 16.92 a | 3.92 a | 12.75 a |  |
| Post-harvest | 12.81 a | 10.24 a | 13.74 a | 13.54 a | 9.18 a | 20.52 a | 20.32 a | 7.18 a | 13.46 a |  |
|  | **Total NDF (g tree^-1^)** | | | | | | | | | |
| Control | 0.00 b | 0.00 b | 0.00 b | 0.00 a | 0.00 b | 0.00 b | 0.00 b | 0.00 b | 0.00 b |  |
| Pre-harvest | 0.01 a | 0.03 a | 1.03 ab | 0.13 a | 0.72 a | 0.60 a | 0.57 a | 0.04 ab | 0.36 ab |  |
| Post-harvest | 0.02 a | 0.04 a | 1.56 a | 0.19 a | 0.72 a | 0.74 a | 0.68 a | 0.07 a | 0.53 a |  |
|  | **NDF allocation (%)** | | | | | | | | | |
| Control | 0.00 b | 0.00 b | 0.00 b | 0.00 b | 0.00 b | 0.00 b | 0.00 b | 0.00 b | 0.00 b |  |
| Pre-harvest | 0.38 a | 1.05 a | 29.20 a | 3.52 a | 20.50 a | 17.56 a | 15.97 a | 1.22 ab | 10.59 a |  |
| Post-harvest | 0.41 a | 0.91 a | 33.01 a | 3.70 a | 16.79 a | 16.55 a | 15.62 a | 1.51 a | 11.50 a |  |

**Supplementary Table 4** Total dry matter content, nitrogen concentration and total N content of separate tree organs harvested at dormancy in June 2018 under the impact of different N fertigation timings. Within each parameter, different letters indicate significant differences (*p* < 0.05) between treatment means (n = 4).

| **Organ type** | **Bud** | **Spur** | **Leaf foliage** | **First-year wood** | **Branch** | **Trunk** | **Old root** | **Fine root** | **Fruit** |  |
| --- | --- | --- | --- | --- | --- | --- | --- | --- | --- | --- |
| **Treatment** | **Total dry matter (g tree^-1^)** | | | | | | | | | |
| Control | 9.18 | 38.5 | 377.16 | 82.76 | 612.51 | 2095.22 | 1593.9 | 102.56 | 4005.8 |  |
| Pre-harvest | 8.92 | 50.23 | 434.78 | 75.5 | 720.53 | 2140.88 | 1599.97 | 144.18 | 5531.6 |  |
| Post-harvest | 10.11 | 49.65 | 347.08 | 74.49 | 728.91 | 1939.37 | 1567.48 | 114.7 | 3448.22 |  |
| 50:50 split | 8.74 | 46.25 | 308.66 | 28.27 | 540.3 | 1341.16 | 1203.94 | 77.94 | 3486.04 |  |
|  | **Nitrogen concentration (% dry weight)** | | | | | | | | | |
| Control | 1.74 | 2.06 | 0.90 | 1.04 | 0.48 b | 0.42 b | 0.83 | 1.03 c | 0.31 |  |
| Pre-harvest | 1.79 | 2.25 | 1.20 | 1.07 | 0.54 ab | 0.50 ab | 0.97 | 1.11 bc | 0.37 |  |
| Post-harvest | 1.83 | 2.12 | 0.90 | 1.21 | 0.67 a | 0.60 a | 0.95 | 1.31 ab | 0.26 |  |
| 50:50 split | 1.88 | 2.04 | 1.22 | 1.25 | 0.51 ab | 0.49 b | 0.99 | 1.34 a | 0.33 |  |
|  | **Total N content (g tree^-1^)** | | | | | | | | | |
| Control | 0.16 | 0.8 | 3.41 | 0.87 | 2.69 b | 8.58 | 13.28 | 1.04 | 11.52 b |  |
| Pre-harvest | 0.16 | 1.13 | 5.3 | 0.8 | 3.83 ab | 10.63 | 15.69 | 1.59 | 20.31 a |  |
| Post-harvest | 0.19 | 1.06 | 3.1 | 0.89 | 4.86 a | 11.66 | 14.85 | 1.50 | 8.99 b |  |
| 50:50 split | 0.17 | 0.95 | 3.81 | 0.33 | 2.68 b | 6.72 | 11.97 | 1.01 | 11.41 b |  |

**Supplementary Table 5** Total dry matter content, nitrogen concentration and total N content of various tree organs harvested at fruit harvest in March 2019 under the impact of different N fertigation timings. Means (n = 4).

| **Organ type** | **Bud** | **Spur** | **Leaf** | **First-year wood** | **Branch** | **Trunk** | **Old root** | **Fine root** | **Fruit** |  |
| --- | --- | --- | --- | --- | --- | --- | --- | --- | --- | --- |
| **Treatment** | **Total dry matter (g tree^-1^)** | | | | | | | | | |
| Control | 9.73 | 16.89 | 601.84 | 89.6 | 1165.43 | 2455.02 | 1800.55 | 116.03 | 998.89 |  |
| Pre-harvest | 8.84 | 15.77 | 534.02 | 93.6 | 1099.48 | 2383.03 | 1325.57 | 110.43 | 1195.72 |  |
| Post-harvest | 10.39 | 21.39 | 663.87 | 126.84 | 1342.98 | 2623.16 | 1453.83 | 119.04 | 1415.29 |  |
|  | **Nitrogen concentration (% dry weight)** | | | | | | | | | |
| Control | 1.38 | 1.92 | 1.53 | 1.14 | 0.65 | 0.41 | 0.59 | 0.76 | 0.24 |  |
| Pre-harvest | 1.38 | 1.88 | 1.59 | 1.13 | 0.69 | 0.42 | 0.69 | 0.85 | 0.25 |  |
| Post-harvest | 1.39 | 1.80 | 1.64 | 1.23 | 0.64 | 0.39 | 0.67 | 0.74 | 0.27 |  |
|  | **Total N content (g tree^-1^)** | | | | | | | | | |
| Control | 0.13 | 0.32 | 9.16 | 1.01 | 7.63 | 10.1 | 10.59 | 0.86 | 2.51 |  |
| Pre-harvest | 0.12 | 0.29 | 8.41 | 1.05 | 7.49 | 9.85 | 9.08 | 0.95 | 2.98 |  |
| Post-harvest | 0.14 | 0.38 | 10.97 | 1.39 | 8.31 | 10.09 | 9.65 | 0.90 | 3.91 |  |
